# Severe cutaneous adverse reactions to drugs: A real-world pharmacovigilance study using the FDA Adverse Event Reporting System database

**DOI:** 10.3389/fphar.2023.1117391

**Published:** 2023-04-04

**Authors:** Dongxuan Li, Jinghui Gou, Jun Zhu, Tongyan Zhang, Feng Liu, Daojun Zhang, Liyang Dai, Wenjun Li, Qinglong Liu, Chunmeng Qin, Qian Du, Songqing Liu

**Affiliations:** ^1^ Department of Pharmacy, The Third Affiliated Hospital of Chongqing Medical University, Chongqing, China; ^2^ College of Pharmacy, Chongqing Medical University, Chongqing, China; ^3^ Infectious Disease Department, Second Affiliated Hospital of Tianjin University of Traditional Chinese Medicine, Tianjin, China; ^4^ Center for Medical Information and Statistics, The Third Affiliated Hospital of Chongqing Medical University, Chongqing, China; ^5^ Department of Dermatology, The Third Affiliated Hospital of Chongqing Medical University, Chongqing, China; ^6^ Medical Data Science Academy, Chongqing Medical University, Chongqing, China

**Keywords:** severe cutaneous adverse reactions, FDA Adverse Event Reporting System, culprit-drug, disproportionality analysis, pharmacovigilance

## Abstract

**Background:** Sound drug safety information is important to optimize patient management, but the widely recognized comprehensive landscape of culprit-drugs that cause severe cutaneous adverse reactions (SCARs) is currently lacking.

**Objective:** The main aim of the study is to provide a comprehensive landscape of culprit-drugs for SCARs to guide clinical practice.

**Methods:** We analyzed reports associated with SCARs in the FDA Adverse Event Reporting System database between 1 January 2004 and 31 December 2021 and compiled a list of drugs with potentially serious skin toxicity. According to this list, we summarized the reporting proportions of different drugs and drug classes and conducted disproportionality analysis for all the drugs. In addition, the risk characteristic of SCARs due to different drugs and drug classes was summarized by the positive–negative distribution based on the results of the disproportionality analysis.

**Results:** A total of 77,789 reports in the FDA Adverse Event Reporting System database were considered SCAR-related, of which lamotrigine (6.2%) was the most reported single drug followed by acetaminophen (5.8%) and allopurinol (5.8%) and antibacterials (20.6%) was the most reported drug class followed by antiepileptics (16.7%) and antineoplastics (11.3%). A total of 1,219 drugs were reported as culprit-drugs causing SCARs in those reports, and the largest number of drugs belonged to antineoplastics. In disproportionality analysis, 776 drugs showed at least one positive pharmacovigilance signal. Drugs with the most positive signals were lamotrigine, acetaminophen, furosemide, and sulfamethoxazole/trimethoprim.

**Conclusion:** Our study provided a real-world overview of SCARs to drugs, and the investigation of SCAR positive–negative distribution across different drugs revealed its risk characteristics, which may help optimize patient management.

## 1 Introduction

Severe cutaneous adverse reactions (SCARs) are relatively uncommon but life-threatening adverse skin reactions, which are caused by an immunologically mediated inflammatory reaction with a prominent phenotype in the skin (Hoetzenecker et al., 2016; Ardern-Jones and Mockenhaupt, 2019; Bellon, 2019). Drug reaction with eosinophilia and systemic symptoms (DRESS), Steven–Johnson syndrome (SJS), toxic epidermal necrolysis (TEN), and acute generalized exanthematous pustulosis (AGEP) are among the most commonly recognized SCARs (Duong et al., 2017). Although SCAR cases are rare, the mortality is relatively high. It is reported that the mortality of all SCARs accounts for 4% for AGEP, 2%–6% for DRESS, and up to 48% for TEN (Owen and Jones, 2021). Due to the very high mortality, the management of SCARs is significantly challenging.

Culprit-drug identification and its early withdrawal are the first mandatory steps for SCAR patients (Paulmann and Mockenhaupt, 2016; Duong et al., 2017; Owen and Jones, 2021; Zhang et al., 2021) because it may decrease mortality (Garcia-Doval et al., 2000; Valeyrie-Allanore et al., 2007). An evidence-based and comprehensive list of causative drugs may aid in the identification and early withdrawal of a culprit-drug, but related work is currently focused on specific drug class, regions, or SCAR subclasses (Kardaun et al., 2013; Oshikoya et al., 2020; Chung et al., 2021; Shukla et al., 2021), which may limit the application of research findings. Therefore, there is an urgent need for studies on the causative drug covering a wide spectrum of drugs in large populations.

Pharmacovigilance is scientific and data gathering activity relating to the detection, monitoring, understanding, and prevention of adverse events (AEs) for a medicine, which is a key component of drug safety regulatory processes and principally involves the identification and evaluation of safety signals associated with the use of a medicinal product (Lucas et al., 2022). Medicine safety monitoring is a continuous and dynamic process throughout all the phases of the life cycle of a drug because although drug safety evaluation is very rigorous and thorough in a pre-clinical trial, these studies are conducted on limited numbers of patients that are selected based on strict eligibility criteria, meaning they do not fully represent real-world populations in limited duration, and it is difficult to detect rare and long-term adverse reactions (ADRs) (Trifirò and Crisafulli, 2022). Therefore, pharmacovigilance is significant in detecting drug ADRs and ensuring medicine safety because it breaks the intrinsic limitations in pre-marketing clinical trials and allows to exhaustively evaluate the drug safety profile by using post-marketing real-world data that are collected during routine clinical care.

Many different approaches are currently applied to achieve medicine safety monitoring in modern pharmacovigilance practice, including spontaneous reporting databases, electronic health record monitoring and research frameworks, social media surveillance, and the use of digital devices (Lavertu et al., 2021). Among those methods, spontaneous reporting database is a kind of well-established platform that is widely used to perform real-world post-marketing studies and provides a real-time overview of major toxicities, thus informing clinical practice for proactive monitoring (Lavertu et al., 2021; Raschi et al., 2021). In this regard, the FDA Adverse Event Reporting System (FAERS) database is a publicly available drug ADR data resource covering the entire population and a wide range of drugs worldwide, with the added advantage of being able to discover these kinds of rare but serious ADRs cost-effectively. Therefore, the FAERS opens a new window for understanding the real-world causative drug of SCARs and provides an unprecedented opportunity to complete the possible causative drug list and risk assessment.

In practice, By probing for disproportionality between drug use and AE occurrence in the FAERS, these real-world AE data can be used to identify the potential culprit-drugs of specific AEs, optimize drug selection for individual patients, and explore drug–drug interaction (Kass-Hout et al., 2016). The present study evaluates the relationship between drugs and SCARs in FAERS using a well-established adverse reaction signal monitoring approach, exploring and summarizing the relationship between drugs and SCARs from the pharmacovigilance perspective and providing a reference for clinical decision-making.

## 2 Methods

### 2.1 Data source

FAERS is a drug adverse reaction information release platform established by using the preferred terms (PTs) of the Medical Dictionary for Drug Regulatory Activities (MedDRA) to code AEs. It currently opens all adverse reaction reports since 2004 in openFDA with more than 10,000,000 patient adverse reaction report data available for public retrieval and downloads and is updated quarterly. These reports include information on patient demographics, medication use, AEs, indications, outcomes, and report sources. By constructing a reasonable query request, the query, screening, statistics, and download of the target adverse reaction report data can be realized through the application programming interface (API) (Kass-Hout et al., 2016). In this study, we reviewed reports on SCARs between 1 January 2004 and 31 December 2021.

### 2.2 Definition of the SCAR report in FAERS

In the study, narrow-scope PTs in the Standardized MedDRA Query (SMQ) were used to identify the target report in FAERS. Within an SMQ, a PT can be classified as having a narrow or broad scope. The narrow scope identifies the PTs that are more likely to represent the condition or an area of interest, while the broad-scope PTs may end up having little to minimal interest for use in the analysis upon further investigation (Mozzicato, 2007). Searching in MedDRA 23.0, there were 18 narrow-scope PTs in the SMQ classification of SCARs (Table 1). If one of those PTs was included in the “patient.reaction.reactionmeddrapt” field of the report, we considered it SCAR-related.

**TABLE 1:** Eighteen narrow-scope PTs in SMQ classification of SCARs.

PT	MedDRA code
Acute generalized exanthematous pustulosis	10048799
Bullous hemorrhagic dermatosis	10083809
Cutaneous vasculitis	10011686
Dermatitis bullous	10012441
Dermatitis exfoliative	10012455
Dermatitis exfoliative generalized	10012456
Drug reaction with eosinophilia and systemic symptoms	10073508
Epidermal necrosis	10059284
Erythema multiforme	10015218
Erythrodermic atopic dermatitis	10082985
Exfoliative rash	10064579
Oculomucocutaneous syndrome	10030081
SJS–TEN overlap	10083164
Skin necrosis	10040893
Stevens–Johnson syndrome	10042033
Target skin lesion	10081998
Toxic epidermal necrolysis	10044223
Toxic skin eruption	10057970
Severe cutaneous adverse reactions (SMQ)^a^	20000020

^a^ This is an SMQ-level term which includes all 18 narrow-scope PTs.

PT, preferred term; MedDRA, Medical Dictionary for Drug Regulatory Activities; SJS, Stevens–Johnson syndrome; TEN, toxic epidermal necrolysis; SMQ, Standardized MedDRA Query.

### 2.3 Adverse reaction signal detection method

Disproportionality analysis was conducted to identify adverse reaction signals by computing the reporting odds ratio (ROR) and the corresponding 95% confidence intervals (CIs). Based on the classic two-by-two contingency table (Table 2), the ROR was calculated as the ratio of the odds of reporting SCARs *versus* all other ADRs for a given drug compared with the reporting odds for all other drugs present in FAERS (Sakaeda et al., 2013). The ROR and 95% CI can be calculated using the following formulas:

$$ROR=\frac{a/c}{b/d}=\frac{ad}{bc},$$ (1)

$$95\%\;CI=e^{\ln\left(ROR\right)\pm 1.96\sqrt{\left(\frac{1}{a}+\frac{1}{b}+\frac{1}{c}+\frac{1}{d}\right)}}.$$ (2)

**TABLE 2:** Two-by-two contingency table for disproportionality analysis.

	Drug of interest	Other drugs	Total
AE of interest	a	b	a + b
Other AEs	c	d	c + d
Total	a + c	b + d	a + b + c + d

AE, adverse event.

When the lower limit of the 95% CI of the ROR was >1 with at least three cases, the ROR was considered significant and regarded as a positive signal (Zhai et al., 2019), which means the drug of interest may have the potential risk to induce the AE of interest. Instead, if the lower limit of the 95% CI and the case number cannot reach the criteria mentioned previously, it means there is a weak association between AE occurrence and drug use, namely, a negative signal.

### 2.4 Data extraction and signal detection

The downloaded file from FAERS through openFDA API is highly structured data stored in “JSON” format. It is a collection of data containing patient demographic and administrative information, the origin of information, drug indication, previous and concurrent medications, dates of commencement and discontinuation of therapy, AEs, and drug use outcome (Altebainawi et al., 2023). By specifying specific fields, we can precisely locate the required information mentioned previously to perform analysis and gather those data into a datasheet. In this study, we used the R packages “httr” to call the API, “jsonlite” to read the downloaded file, and “dplyr” to sort out and analyze the data. The specific execution process is as follows.

First, we downloaded all SCAR-related report data between 1 January 2004 and 31 December 2021 from the FAERS database through API and extracted basic report information, including report time, report source, patient demographic information, adverse event outcomes, and the number of reports involving each PT.

Second, we extracted the drug information from the downloaded dataset. According to the reported role of the drug in the report, drugs can be divided into primary suspect drugs, concomitant drugs, and drugs interacting with the suspect drug. For the accurate collection of culprit-drugs, only primary suspect drugs (“patient.drug.drugcharacterization” field = 1) were reserved. After obtaining the primary suspect drug generic names in the “patient.drug.openfda.generic_name” field on all SCAR reports, to obtain the final culprit-drug list for the signal detection, we excluded drugs that were missing generic names, duplicated, and ambiguous.

Third, the Anatomical Therapeutic Chemical (ATC) system was used to categorize drugs into classes according to their therapeutic effects and characteristics (Skrbo et al., 2004). For drugs with the same active ingredient (e.g., different salt forms), professional pharmacists performed manual integration.

Fourth, 18 narrow-scope PTs combined with the drug were used to compute ROR and 95% CI. After that, the total ROR and 95% CI were calculated for each drug using the SMQ level, so there are 19 ROR and 95% CI values (one for the SMQ level and 18 for the PT level) for each evaluated drug.

Fifth, according to the PT subclass and SMQ level, we conducted categorical statistics on the SCAR reports in FAERS, yielding the top 10 agents and ATC drug classes (second ATC level) with the highest reporting proportions.

Finally, according to the signal detection results, the drugs were divided into positive signal and negative signal drugs. To achieve the final list of drugs closely related to SCARs, drugs with at least one of the 19 signals that met the criteria for a positive signal were screened.

In this study, R version 4.1.0 (R Foundation for Statistical Computing, Vienna, Austria) was used for the data processing and analysis described previously.

## 3 Results

### 3.1 Basic information and patient characteristics according to the report

**FIGURE 1:**
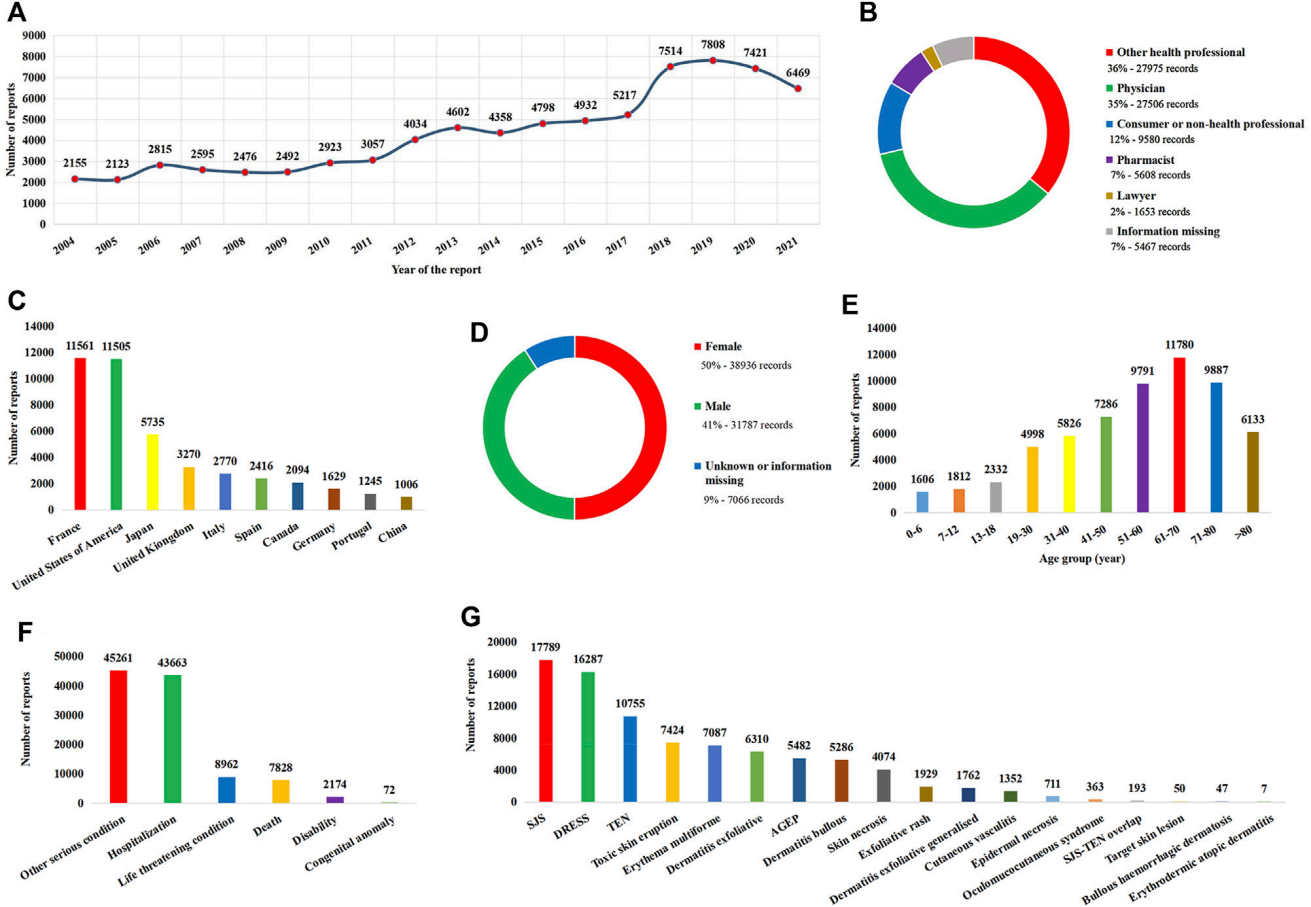
Basic information and patient characteristics according to the report. **(A)** Distribution of the reporting year. **(B)** Distribution of the reporter. **(C)** Top 10 countries with the most sources of reports. **(D)** Distribution of patient gender. **(E)** Distribution of patient age. **(F)** Distribution of the patient outcome. **(G)** Distribution of adverse reactions of preferred terms. AGEP, acute generalized exanthematous pustulosis; DRESS, drug reaction with eosinophilia and systemic symptoms; SJS, Stevens–Johnson syndrome; TEN, toxic epidermal necrolysis.

A total of 14,496,963 reports were included in FAERS between 1 January 2004 and 31 December 2021. Of these, 77,789 (0.54%) reports were considered SCAR-related. Reports on SCARs have been increasing in recent years, with 2019 being the year with the most reports (Figure 1A). In terms of report submission, health professionals (78%) were the leading reporters (Figure 1B), and France and the United States were the leading reporting countries (Figure 1C). In terms of patients, there were more female patients than male patients (Figure 1D), and the main age group was 61–70 years (Figure 1E). Furthermore, SCARs can lead to severe outcomes, accounting for 7,828 (10%) patient deaths (Figure 1F). Among the narrow-scope PTs included in SCARs, SJS was the most reported PT (Figure 1G).

### 3.2 Identification of the culprit-drug list

**FIGURE 2:**
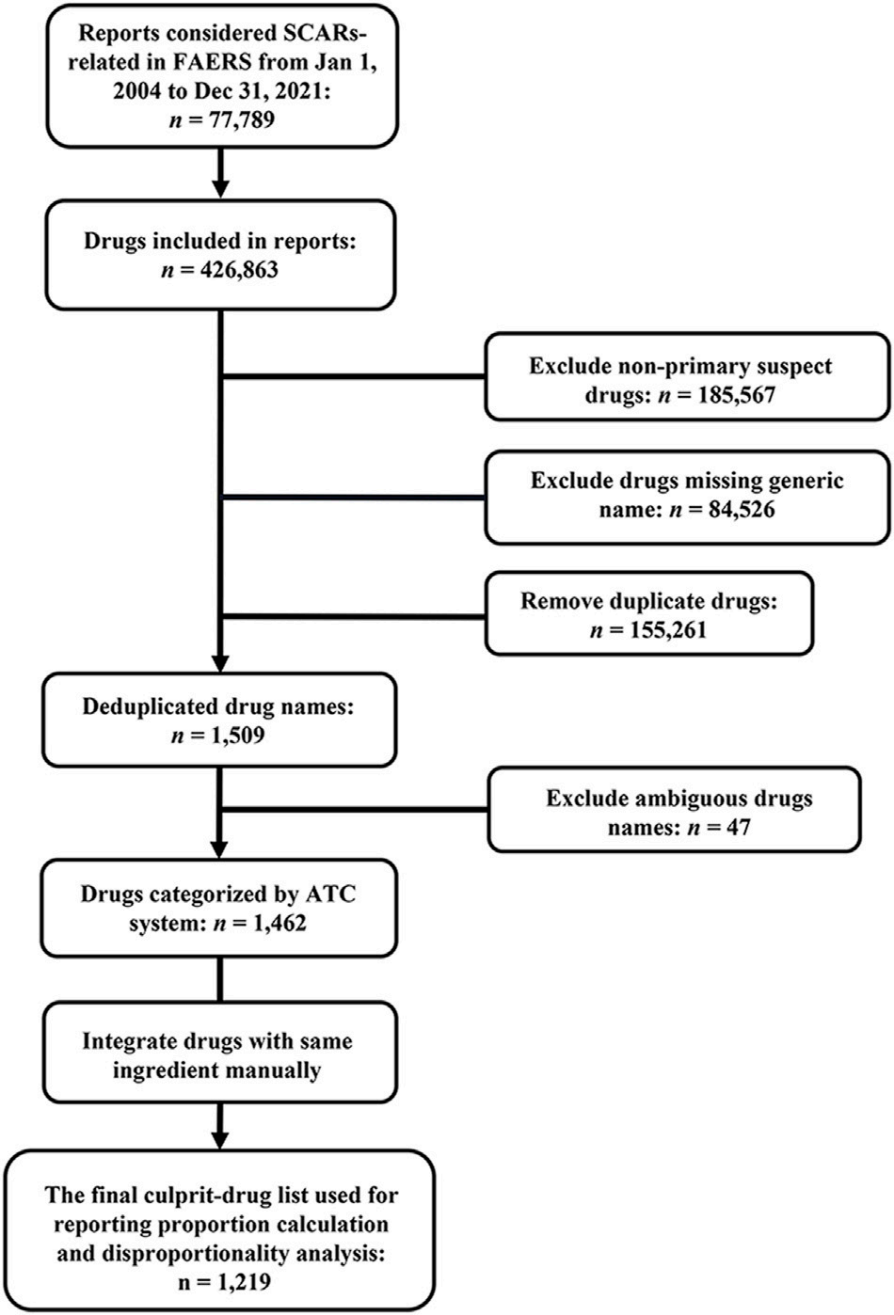
Flowchart for data acquisition and processing. ATC, Anatomical Therapeutic Chemical; FAERS, FDA Adverse Event Reporting System; SCARs, severe cutaneous adverse reactions.

As each report usually included multiple drugs, there were a total of 426,863 drugs recorded in 77,789 SCAR-related reports. Due to reporters who were mainly health professionals who had preliminarily evaluated the role of drugs in the development of SCARs, primary suspect drugs were included in the analysis as a culprit-drug in this study. After excluding non-primary suspect drugs, drugs that were missing generic names, duplicated, and ambiguous, and integrating drugs with the same active ingredient, a total of 1,219 drugs made up the final culprit-drug list, which means each drug in the list was classified as primary suspected drugs in at least one report (Figure 2). However, the number culprit-drugs varied across PTs, with the SJS group (80.5%) containing the most drugs and the EAD group (1.4%) containing the least (Table 3).

**TABLE 3:** Number of culprit-drugs of the SMQ group and each PT subgroup.

Group	No. (%) of drugs among 1,219 culprit-drugs
Stevens–Johnson syndrome	981 (80.5)
Erythema multiforme	864 (70.9)
Toxic epidermal necrolysis	852 (69.9)
Dermatitis exfoliative	839 (68.8)
Dermatitis bullous	812 (66.6)
Drug reaction with eosinophilia and systemic symptoms	804 (66.0)
Toxic skin eruption	786 (64.5)
Skin necrosis	744 (61.0)
Exfoliative rash	680 (55.8)
Acute generalized exanthematous pustulosis	628 (51.5)
Dermatitis exfoliative generalized	525 (43.1)
Cutaneous vasculitis	497 (40.8)
Epidermal necrosis	375 (30.8)
Oculomucocutaneous syndrome	154 (12.6)
SJS–TEN overlap	113 (9.3)
Bullous hemorrhagic dermatosis	68 (5.6)
Target skin lesion	29 (2.4)
Erythrodermic atopic dermatitis	17 (1.4)
Severe cutaneous adverse reactions (SMQ)^a^	1,219 (100.0)

^a^ This is an SMQ term which includes all 18 narrow preferred terms.

MedDRA, Medical Dictionary for Drug Regulatory Activities; SJS, Stevens–Johnson syndrome; TEN, toxic epidermal necrolysis; SMQ, Standardized MedDRA Query.

### 3.3 Reporting proportions of culprit-drugs

**FIGURE 3:**
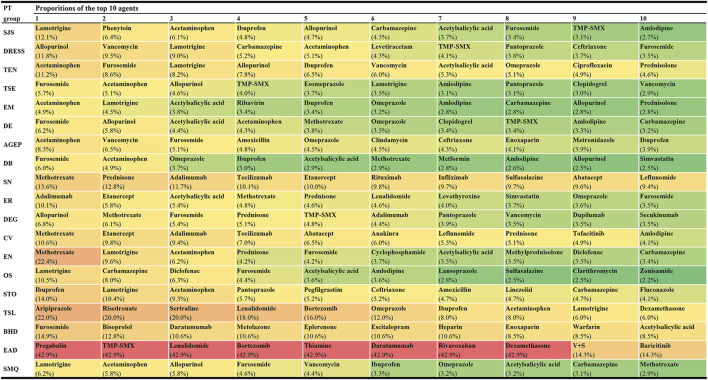
Top 10 agents with the highest reporting proportions at the SMQ and PT levels. AGEP, acute generalised exanthematous pustulosis; BHD, bullous haemorrhagic dermatosis; CV, cutaneous vasculitis; DB, dermatitis bullous; DE, dermatitis exfoliative; DEG, dermatitis exfoliative generalized; DRESS, drug reaction with eosinophilia and systemic symptoms; EN, epidermal necrosis; EM, erythema multiforme; EAD, erythrodermic atopic dermatitis; ER, exfoliative rash; OS, oculomucocutaneous syndrome; PT, preferred term; SCARs, severe cutaneous adverse reactions; SMQ, Standardized MedDRA Queries; STO, SJS-TEN overlap; SN, skin necrosis; SJS, Stevens-Johnson syndrome; TMP-SMX, sulfamethoxazole and trimethoprim; TSL, target skin lesion; TEN, toxic epidermal necrolysis; TSE, toxic skin eruption; V+S, velpatasvir and sofosbuvir.

**FIGURE 4:**
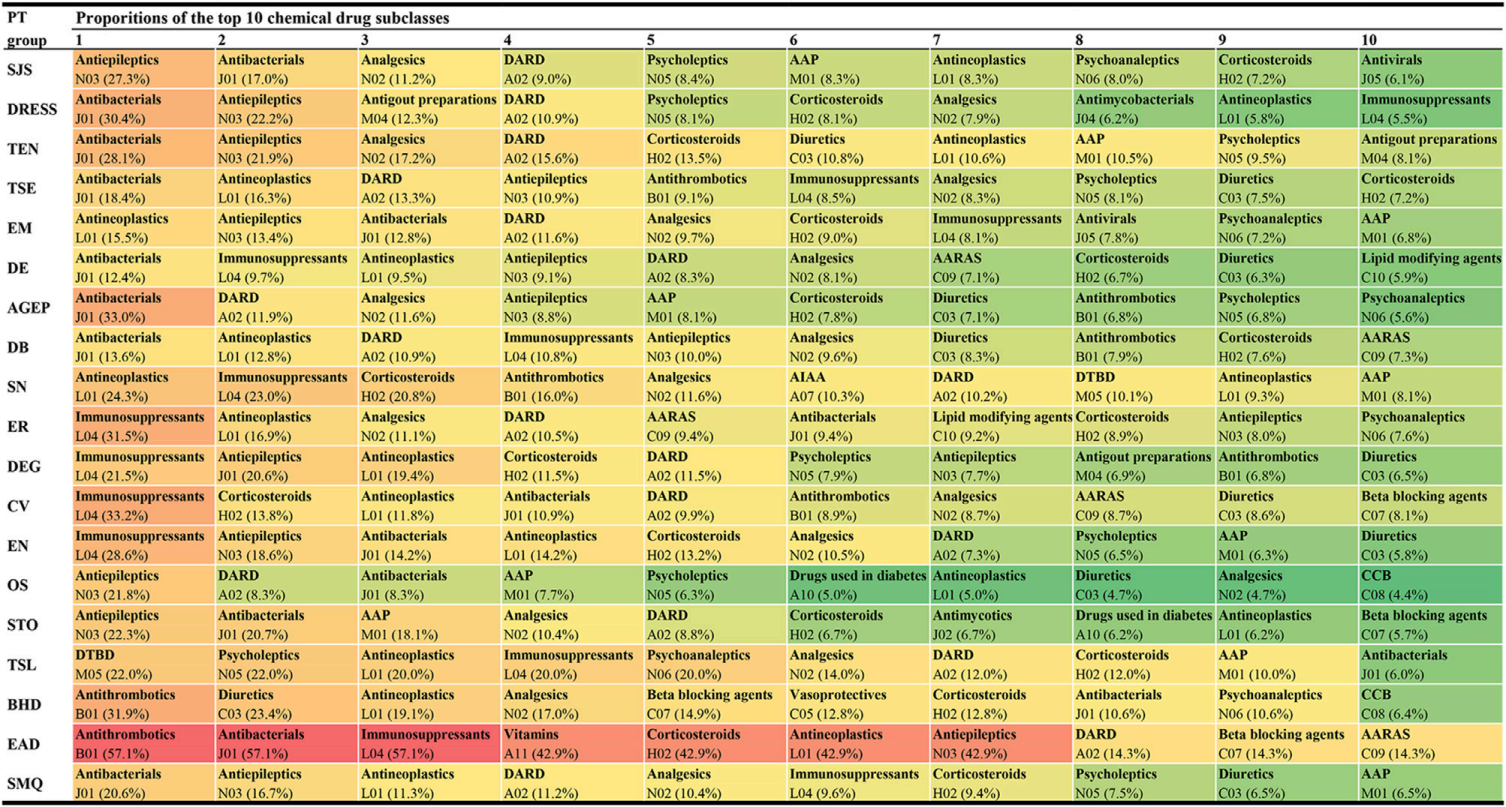
Top 10 drug classes with the highest reporting proportions at the SMQ and PT levels. AAP, antiinflammatory and antirheumatic products; AARAS, agents acting on the renin-angiotensin system; AIAA, antidiarrheals or intestinal antiinflammatory/antiinfective agents; AGEP, acute generalised exanthematous pustulosis; BHD, bullous haemorrhagic dermatosis; CCB, calcium channel blockers; CV, cutaneous vasculitis; DARD, drugs for acid related disorders; DB, dermatitis bullous; DE, dermatitis exfoliative; DEG, dermatitis exfoliative generalized; DRESS, drug reaction with eosinophilia and systemic symptoms; DTBD, drugs for treatment of bone diseases; EN, epidermal necrosis; EM, erythema multiforme; EAD, erythrodermic atopic dermatitis; ER, exfoliative rash; OS, oculomucocutaneous syndrome; PT, preferred term; SCARs, severe cutaneous adverse reactions; SMQ, Standardized MedDRA Queries; STO, SJS-TEN overlap; SN, skin necrosis; SJS, Stevens-Johnson syndrome; TSL, target skin lesion; TEN, toxic epidermal necrolysis; TSE, toxic skin eruption.

Among these 1,219 kinds of drugs, the top 10 agents with the highest reporting proportions at the SMQ and PT levels are presented in Figure 3. According to the ATC classification (second level), the top 10 drug classes with the highest reporting proportions at the SMQ and PT levels are presented in Figure 4.

### 3.4 Adverse reaction signal detection results

Each potential culprit-drug causing SCARs was combined with SMQ and each PT for disproportionality analysis, yielding 19 pharmacovigilance signals for each drug. Details of the disproportionality analysis results for each drug are listed in Supplementary Table S1.

**FIGURE 5:**
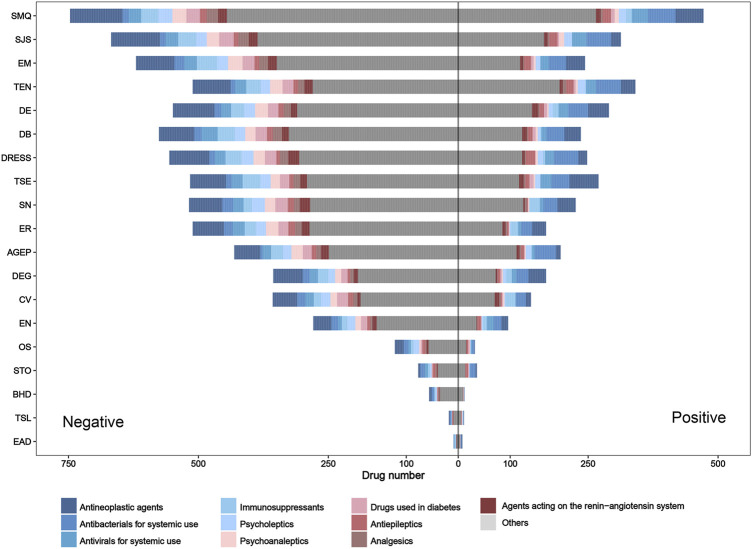
Pharmacovigilance signal distribution and drug class distribution of reported culprit-drugs at the SMQ and PT levels. AGEP, acute generalized exanthematous pustulosis; BHD, bullous hemorrhagic dermatosis; CV, cutaneous vasculitis; DB, dermatitis bullous; DE, dermatitis exfoliative; DEG, dermatitis exfoliative generalized; DRESS, drug reaction with eosinophilia and systemic symptoms; EN, epidermal necrosis; EM, erythema multiforme; EAD, erythrodermic atopic dermatitis; ER, exfoliative rash; OS, oculomucocutaneous syndrome; PT, preferred term; SMQ, Standardized MedDRA Query; STO, SJS–TEN overlap; SN, skin necrosis; SJS, Stevens–Johnson syndrome; TSL, target skin lesion; TEN, toxic epidermal necrolysis; TSE, toxic skin eruption.

For the SMQ level and each PT, the distribution of adverse drug reaction signals and drug classes is presented in Figure 5. On the whole, the number of positive signal drugs in each group was less than that of negative signal drugs. In terms of the drug class (ATC second level), antineoplastic agents, antibacterials for systemic use, and antivirals for systemic use were the top three drug categories involved in most groups. However, it is worth noting that antibacterials for systemic use showed the largest rate of positive drugs in most PT groups.

Due to the overlap and differences in positive drugs in different groups, to obtain drugs with a strong statistical association with SCARs, we integrated a positive signal number for each drug (Table 4). Of the 1,219 drugs, the number of positive signals for each drug is between 0 and 16, and 776 drugs had at least one positive signal in each group. Among these drugs, four drugs, namely, lamotrigine, acetaminophen, furosemide, and sulfamethoxazole/trimethoprim, contained the most positive signals, and each drug included 16 positive signals (Supplementary Table S1).

**TABLE 4:** Distribution of the number of positive adverse drug reaction signals.

Positive ADR signal number	No. (%) of culprit-drugs
16	4 (0.3)
15	9 (0.7)
14	9 (0.7)
13	9 (0.7)
12	21 (1.7)
11	14 (1.1)
10	26 (2.1)
9	21 (1.7)
8	36 (3.0)
7	48 (3.9)
6	47 (3.9)
5	63 (5.2)
4	75 (6.2)
3	90 (7.4)
2	118 (9.7)
1	186 (15.3)
0	443 (36.3)
Total	1,219 (100.0)

ADR, adverse drug reaction.

## 4 Discussion

SCARs is an interdisciplinary clinical problem that has received extensive attention from both dermatologists and pharmacists. In this study, we provided a comprehensive overview of drugs causing SCARs from the pharmacovigilance perspective, summarized a list of 1,219 drugs that were reported as culprit-drugs causing SCARs in FAERS between 2004 and 2021, and described the reporting proportions of different drugs and drug classes. At the same time, we further detected the risk signals of the aforementioned drugs and evaluated the risk association between the occurrence of SCARs and drugs. To our knowledge, this study provides, for the first time, a comprehensive list of SCAR-causing drugs and shows a novel strategy to gain a comprehensive understanding of SCAR-causing drugs. Although the intrinsic nature of spontaneous reporting databases leads to unavoidable limitations in the application of our results, if we use those results properly, it can help to some extent in the rapid and evidence-based identification of culprit-drugs causing SCARs in the clinical setting.

In this study, we offered a multi-dimensional evaluation perspective. First, we presented the top 10 agents and drug classes with the highest reporting proportions at the SMQ and PT levels, respectively. Through this result, we can understand the drugs and drug classes that commonly cause SCAR in the clinic and can further locate specific subtypes of SCARs, such as SJS, TEN, DRESS, or AGEP. In our study, lamotrigine (SMQ level) was the agent with the highest reporting proportion followed by acetaminophen and allopurinol, while antibacterials (SMQ level) were the drug class with the highest reporting proportions followed by antiepileptics and antineoplastic agents. However, our results are not consistent with previous studies in reporting the proportion rank (Kardaun et al., 2013; Su and Aw, 2014; Zhao et al., 2019; Oshikoya et al., 2020; Ambe et al., 2021). Reporting on the proportions of drugs or drug classes may vary according to the region, study design, sample size, and patient inclusion and exclusion criteria, but our larger sample and global perspective make our results on reporting proportions more reliable, giving more precise guidance on which drugs to focus on. However, we also noted that higher reporting proportions do not necessarily mean a higher risk of SCARs because differences in the frequency of drug use may also lead to a higher reporting proportion.

For the aforementioned reason, in this study, we introduced the pharmacovigilance method as a more uniform metric for risk assessments to evaluate the ADR signals of each drug at the SMQ and PT levels. Although there have been studies using signal mining methods to assess the risk of drugs causing SCARs, the scope of the research is limited to specific drug classes or SCAR subclasses (Xu et al., 2021; Zhu et al., 2021; Bomze et al., 2022), so that the risk comparison between drugs can only be achieved within a limited range. In this study, we extended this scope to all reported culprit-drugs to date, allowing for cross-drug class, cross-PT risk assessment (Supplementary Table S1), which is more applicable to rapidly assess the risk of drugs causing SCARs in clinical practice. In addition, we summarized the distribution of pharmacovigilance signals, with 776 of the 1,219 drugs showing at least one positive signal, suggesting that those drugs need more attention in clinical use, especially drugs that exhibit multiple positive signals, such as lamotrigine, acetaminophen, furosemide, and sulfamethoxazole/trimethoprim. If we apply these results rationally, it will help optimize culprit-drug identification and the early withdrawal of them from treatment.

In addition, it is noteworthy that, of the 1,219 drugs, antineoplastics were the drug class containing the largest number of drugs and the drug class with the third highest reporting proportion (SMQ level) in FAERS. In previous studies, antineoplastics were not a common culprit-drug causing SCARs (Kardaun et al., 2013; Su and Aw, 2014; Zhao et al., 2019; Oshikoya et al., 2020), but our results suggested that anti-tumor drugs play an important role in the occurrence of SCARs. We believe that the difference should be mainly attributed to the development and clinical application of novel anti-tumor drugs and widespread attention to their skin toxicity in recent years (Quach et al., 2021; Nikolaou et al., 2022). In real-world scenarios, pharmacovigilance can be a handy tool for detecting and validating the potential skin toxicity of drugs, and several studies using the pharmacovigilance method have reported a risk of SCARs caused by some targeted antineoplastic drugs (Yang et al., 2021; Zhu et al., 2021), and some of the results were verified in clinical practice (Birmingham et al., 2022; Oya et al., 2022). Our results are consistent with their data mining findings, which illustrates the reliability of our results, more importantly; however, we captured that the main causative drugs of SCARs have changed in recent years, and antineoplastics have become an important drug class leading to SCARs. Capturing such changes will help find drugs with a focus on the original basis, which may optimize the management of SCARs. In this regard, our study provides an opportunity and strategy to capture such changes.

The present study also has some unavoidable limitations. First, due to the voluntary nature of reporting to FAERS, the fact that some data was not peer-reviewed may bias the results. A high number of reported cases for SCARs in the FAERS database were from healthcare providers (78%), which may improve the quality of reporting. Second, the actual incidence of SCARs due to drugs cannot be determined, as the total number of patients using these medications is unknown. Third, the signal detection results only suggest that there is a statistical association, and the question of whether there is a real causal relationship still needs further evaluation. Fourth, we cannot eliminate the potential influence of the presence of concomitant therapeutic drugs and (or) comorbidities on the occurrence of SCARs, which may bias our signal detection results. Fifth, underreporting, Weber effect, and notoriety bias may exist in some drugs or drug classes, and may lead to a biased result, but recent studies have shown that these factors have relatively little effect on FAERS (Hoffman et al., 2014; Neha et al., 2021). In this study, we did not evaluate the influence of these factors on our research results, so their influence on our results is still unknown. Finally, our study can only provide a reference for the culprit-drug identification of SCARs and cannot replace the professional opinions of dermatologists.

## 5 Conclusion

The large population, wide geographic coverage, and publicly available accessibility of FAERS have qualified this spontaneous ADR reporting data source as an important resource in the study of the culprit-drug landscape of SCARs. As the largest study of its kind, we provided a whole picture of SCARs in a worldwide landscape. Our study provides evidence that can help to quickly identify the culprit-drugs that might cause SCARs. It may be relevant to many interested parties, including regulators, medical personnel, and others involved in drug management and use. Meanwhile, our work provides a powerful strategy to mine for information on drugs related to SCARs in the future and provides a real-world window for developing a pharmacovigilance strategy for drug-related injuries. However, it is particularly noteworthy that our studies, as a pharmacovigilance study using FAERS, can only provide a signal of possible associations between drugs and ADRs, so it is still necessary to conduct further investigation through appropriate research to verify the true relationship between drugs and ADRs.

## Data Availability

The original contributions presented in the study are included in the article/Supplementary Material; further inquiries can be directed to the corresponding authors.
